# Determination of Tritium Transfer Parameters in Lithium Ceramics Li_2_TiO_3_ During Reactor Irradiation Based on a Complex Model

**DOI:** 10.3390/ma18174117

**Published:** 2025-09-02

**Authors:** Timur Zholdybayev, Timur Kulsartov, Zhanna Zaurbekova, Yevgen Chikhray, Asset Shaimerdenov, Magzhan Aitkulov, Saulet Askerbekov, Inesh Kenzhina, Assyl Akhanov, Alexandr Yelishenkov

**Affiliations:** 1 Institute of Nuclear Physics, Almaty 050032, Kazakhstan; zholdybayev@inp.kz (T.Z.); zzha@physics.kz (Z.Z.); ashaimerdenov@inp.kz (A.S.); maitkulov@inp.kz (M.A.); askerbekov@physics.kz (S.A.); kenzhina@physics.kz (I.K.); aakhanov@inp.kz (A.A.); aleksandrelisenkov282@gmail.com (A.Y.); 2 Department of General Physics, Satbayev University, Almaty 050000, Kazakhstan; chikhray@physics.kz; 3 Institute of Experimental and Theoretical Physics, Al-Farabi Kazakh National University, Almaty 050040, Kazakhstan; 4 Al-Farabi Kazakh National University, Almaty 050040, Kazakhstan; 5 Advanced Electronics Development Laboratory, Kazakh-British Technical University, Almaty 050035, Kazakhstan

**Keywords:** tritium, diffusion, lithium ceramics, Li_2_TiO_3_, reactor irradiation, modeling, diffusion coefficient

## Abstract

This paper presents the results of determining the parameters of tritium transfer processes in lithium ceramics Li_2_TiO_3_ under reactor irradiation conditions. Analysis of sections with a short-term decrease in reactor power allowed numerical determination of the Arrhenius parameters of tritium diffusion (pre-exponential factor and activation energy) based on comparison with in situ experimental data. The obtained values of activation energy (70.2–74.7 kJ/mol) and pre-exponential factor (0.9–2.1 × 10^−8^m^2^/s) demonstrate growth with increasing fluence, which is explained by the accumulation of radiation defects in ceramics. A linear dependence was established between *D*_0_ and *E_a_*, corresponding to the Mayer–Noldel rule. Unlike previously conducted studies based on a phenomenological approach to assessing only the activation energy of diffusion, in this study, a complex model that takes into account temperature gradients, tritium generation, its diffusion, and release from the surface was used. The applicability of such an integrated approach to the analysis of in situ reactor experiments with lithium ceramics was confirmed, and allowed us to estimate changes in the tritium transfer parameters in lithium ceramics Li_2_TiO_3_ depending on the irradiation time.

## 1. Introduction

Lithium oxide ceramics, particularly lithium metatitanate (Li_2_TiO_3_), are among the most promising breeder-zone materials for fusion reactor blanket modules. Their high chemical stability, low activation level, favorable thermal properties, and efficient tritium release at moderate temperatures make Li_2_TiO_3_ key candidates for long-term operation under intense neutron irradiation in fusion reactors [1,2,3,4,5].

The primary task in studying lithium ceramics is experimental determination of tritium generation, migration, and release parameters caused by the following nuclear reaction:

(1) Li6+n→He4+T+4.8 MeV.

In this regard, in situ studies are especially valuable, as they measure the flux of released tritium directly during reactor irradiation of ceramic samples, providing realistic operational data on the processes occurring within the material.

To date, numerous reactor tests of lithium ceramics have been performed at leading research centers: the EXOTIC experiments in the HFR reactor (Petten, The Netherlands) [6,7,8,9], a series of tests in the JMTR reactor (Japan) [10,11], and in situ observations at the WWR-K reactor (Kazakhstan) and other complexes [12,13,14,15,16,17,18,19]. These experiments employed both temperature cycling and vacuum conditions to assess desorption kinetics, trapping and enrichment effects, and tritium distribution within the ceramic.

Most models used to analyze reactor experiments rely either on an effective diffusion coefficient approximation or on simplified one-dimensional schemes. In this case, spatial temperature gradients, arising from samples and the irradiation capsule geometry, as well as heat transfer and radiation exchange processes in the “sample-ampoule” system, are seldom taken into account.

Nevertheless, a number of works present complex numerical models describing the behavior of tritium in lithium ceramics, taking into account the coupled processes of generation, diffusion, heat transfer, and desorption. Thus, in [20], a model of tritium transport in lithium metatitanate taking into account radiation-induced defects affecting the diffusion coefficient during irradiation is presented. The study [21] implements two-dimensional numerical modeling of tritium transport taking into account thermal gradients, desorption from the surface, and geometric features of spherical granules. In the work [22], a model is proposed that includes sorption-desorption processes and diffusion in porous ceramics. It is important to note that, unlike previously proposed models such as analytical TPD approaches [23], thermal gradient-free modeling [24], blanket-level system CFD models [25], and reactor-independent pore models [22], the model considered in this paper is aimed at direct reproduction of experimental data taking into account the specific geometry of the pebble.

Earlier in [14], the authors of the present paper proposed a phenomenological method for estimating the activation energy of tritium diffusion in lithium ceramics by analyzing experimental intervals during which reactor power was briefly reduced. It was assumed that over a short period, temperature changes significantly affect the diffusion coefficient, while the tritium concentration gradient remains nearly constant. The variation in the tritium production rate during a short reactor power reduction was also neglected. This approach enabled the estimation of the diffusion activation energy, but it became clear that these experimental intervals require more accurate analysis, considering the changes in tritium generation and diffusion throughout the sample by solving three-dimensional transport equations and modeling the temperature field in the ceramics.

The present work focuses on a more precise analysis of such experimental sections, using a previously developed complex model, which now incorporates the following:

- Temperature distribution within the entire volume of lithium ceramics, considering the decrease in energy release as reactor power varies and the actual geometry of the contact between the sample and the capsule.
- Changes in tritium generation rate in the sample under short-term reactor power reduction.
- Diffusive transport with a temperature-dependent diffusion coefficient.

This approach allows numerical determination of the Arrhenius parameters (pre-exponential factor and activation energy) for the tritium diffusion coefficient by comparing the calculated and experimentally measured tritium fluxes from lithium ceramic samples. Thus, for the first time, real in-situ experimental data are combined with complex simulations under conditions of a short-term reactor power decrease.

## 2. Materials and Methods

### 2.1. Experimental Part

A detailed description of the simulated experiment was provided earlier in [12,13,14,15]. These studies were performed at the WWR-K research reactor in Almaty city, Kazakhstan, using the CIRRA (Complex of In-Reactor Release Analysis) experimental facility. Spherical Li_2_TiO_3_ ceramic samples enriched to approximately 96% in the ^6^Li isotope served as the test specimens. Each pebble had a diameter of 1 mm and was fabricated via the indirect wet-process method [26].

The samples were placed in the lower section of a loading capsule installed in the reactor ampoule device and connected to a vacuum-pumping and registration system. Irradiation was carried out under high vacuum conditions, with a residual pressure in the measuring chamber of less than 1.3 × 10^−4^ Pa. Thermocouples positioned in the sample region recorded temperature data throughout the irradiation. According to [13,19], tritium in Li_2_TiO_3_ is predominantly released in the form of HT, with its flux decreasing over time and T_2_ increasing. During steady-state irradiation, the release is stable. Helium, on the other hand, causes short-term peaks in the release at the beginning of irradiation, associated with the opening of pores, while tritium diffuses uniformly even from closed areas.

Regarding the experiment features, short-term (up to 10 min) reductions of the reactor power by 25–50% were performed periodically (~1–2 times a day) during the irradiation, followed by restoration. These shifts caused a quick drop in the sample’s temperature and a corresponding decrease in the flow of tritium-containing molecules (mainly HT).

Previously, within the framework of the phenomenological approach, sections of the experiment with power changes were used for an approximate estimate of the activation energy of tritium diffusion [14]. In this study, these sections will be used for numerical modeling of tritium transfer processes in ceramics, employing a complex model that allows for a more accurate consideration of thermal and transfer processes within the sample volume.

### 2.2. Description of the Model

For the numerical analysis of the processes involving the generation, transfer, and release of tritium in lithium ceramics Li_2_TiO_3_ under reactor irradiation conditions, a previously developed complex model implemented in the COMSOL6 Multiphysics environment [18] was used. The model includes a conjugate solution for heat transfer, tritium production, its diffusion within the ceramic volume, and desorption from the surface. This formulation of the problem enables us to describe the behavior of tritium in a non-uniform temperature field caused by local heat exchange features and the geometry of the samples.

**Figure 1:**
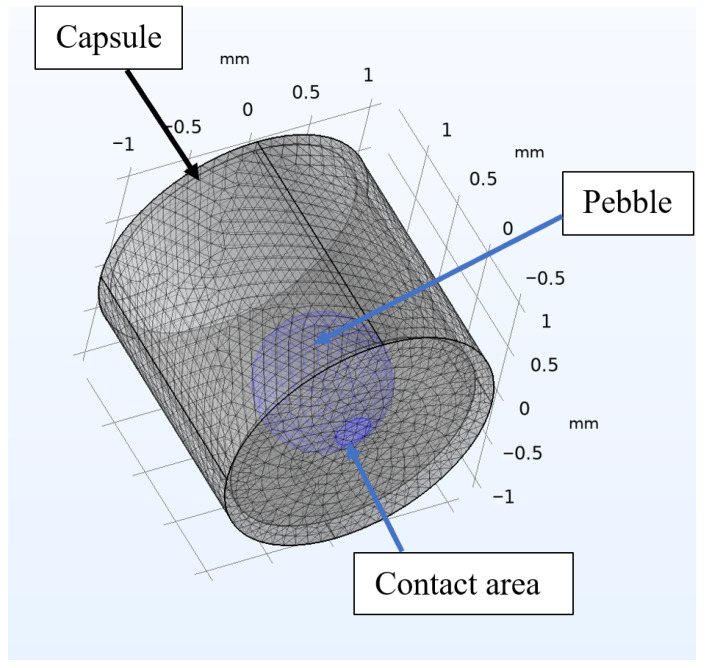
Geometry of the model.

Let us briefly describe the developed model, which is presented in detail in [18]. The object being modeled is a spherical ceramic sample with a radius of 0.5 mm, positioned on the flat surface of a metal capsule (Figure 1).

In thermal calculations, the heat conduction equation with an internal heat source was solved, considering a contact zone between the sample and the base of the capsule, which is a circle with a diameter of 0.04 mm. Additionally, heat exchange by radiation between the ceramic surface and the capsule is included.

The transport of tritium within the ceramic volume in the developed model is described by the diffusion equation, considering the source of diffusant generation:

(2) $$\frac{\partial C(\bar{r},t)}{\partial t}-\frac{D}{{\bar{r}}^{2}}\cdot \frac{\partial }{\partial \bar{r}}({\bar{r}}^{2}\frac{\partial C\left(\bar{r},t\right)}{\partial \bar{r}})=F$$

where $\bar{r}$ is a variable radius vector; *C* is the tritium concentration; *F* is the tritium generation rate; *D* is the diffusion coefficient with Arrhenius temperature dependence:

(3) $$D\left(T(t)\right)=D_{0}\cdot exp\left(-\frac{E_{D}}{RT\left(t\right)}\right).$$

On the surface of the sample, the boundary condition for the diffusant (tritium) is set to zero concentration, which aligns well with the vacuum pumping mode. Desorption at temperatures above 500 °C (the range used in the modeling) is not a limiting factor for its release. Therefore, we can adopt the above boundary condition:

(4) $$C\left(R,t\right)=0,$$

where *R* is the radius of the sample.

It is worth noting that in [18], where the initial release of tritium was modeled during the increase in reactor power, the boundary condition was set by defining the desorption flow. This was necessary to describe the released tritium flow over a wide temperature range, from 20 °C to 650 °C. Consequently, modeling the desorption processes was required for the low-temperature segment.

The tritium flux from the sample is calculated using Fick’s second law. The initial tritium concentration in the pebble before a power change was determined by solving the model equations for the sample’s holding time of approximately 160,000 s (~1.85 days) at 6 MW of reactor power. This method enabled us to determine the volumetric tritium concentration in the pebble at the equilibrium state of tritium production and release processes.

The tritium transfer parameters in lithium ceramics were determined by comparing the results of tritium flux calculations with the experimentally recorded normalized dependence of the tritium flux from the sample. The simulation was conducted on experimental segments where a short-term change in reactor power occurred, resulting in changes in sample temperature and the fluxes of tritium-containing molecules.

### 2.3. Input Parameters of the Model

The following functions were included as inputs in the model:

*W*(*t*)—change in reactor power in specific segments of the experiment. It was used to correct the time dependencies of the volumetric energy release in the sample and tritium generation rate.

*T*(*t*)—experimental data on the ampoule bottom temperature. These data were used to determine how the temperature field changes over the volume of ceramics in the selected experimental sections. It was then used to calculate the diffusion coefficients using the model’s diffusion equations.

$J_{T}(t)$—experimental data on the tritium flux leaving the sample:

(5) $$J_{T}(t)=J_{HT}(t)+2J_{T_{2}}(t)$$

During the calculations, two main parameters of the temperature dependence of the tritium diffusion coefficient were varied: the pre-exponential factor *D*_0_ and the activation energy *E_D_*.

To evaluate the agreement between the calculated and experimental tritium release curves, the residual function was used:

(6) $$\delta =\sum \left|J_{model}(t)-J_{T}\left(t\right)\right|$$

The search for optimal parameters was simplified to locating the minimum of the function *δ*, a similar approach to that used in the calculations presented in [18]. Therefore, the diffusion parameters were identified as those that provide the best quantitative match between the whole model curve and the experimental data under varying reactor power conditions.

## 3. Simulation Results

**Figure 2:**
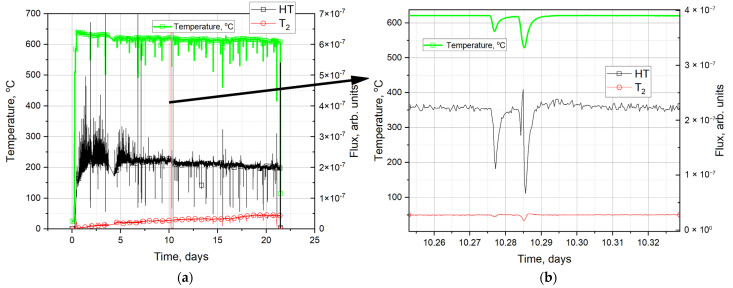
(**a**) Diagram showing changes in sample temperature, HT, and T_2_ fluxes during the experiment. (**b**) Including a characteristic section of a short-term decrease in reactor power.

From the general diagram of experiments (Figure 2), five sections were selected in which a short-term decrease in power was implemented (Table 1). Then, for each section, modeling was performed to determine the optimal diffusion parameters that best fit the experimental curves in each section.

For the first section, the simulation was conducted 56 times with different sets of parameters: in the range of 66–80 kJ/mol for activation energy *E_D_* and (10^−9^–6 × 10^−8^) m^2^/s for the diffusion coefficient *D*_0_. These variation ranges were chosen based on the diffusion coefficient data obtained from the initial part of the experiment [18].

The trends of the *δ* function change during the modeling process were analyzed, and gradual improvements of the variation intervals for the parameters of the tritium diffusion coefficient in ceramics were performed. As a result, for each remaining section of the experiment, the modeling was conducted no more than 20 times.

**Figure 3:**
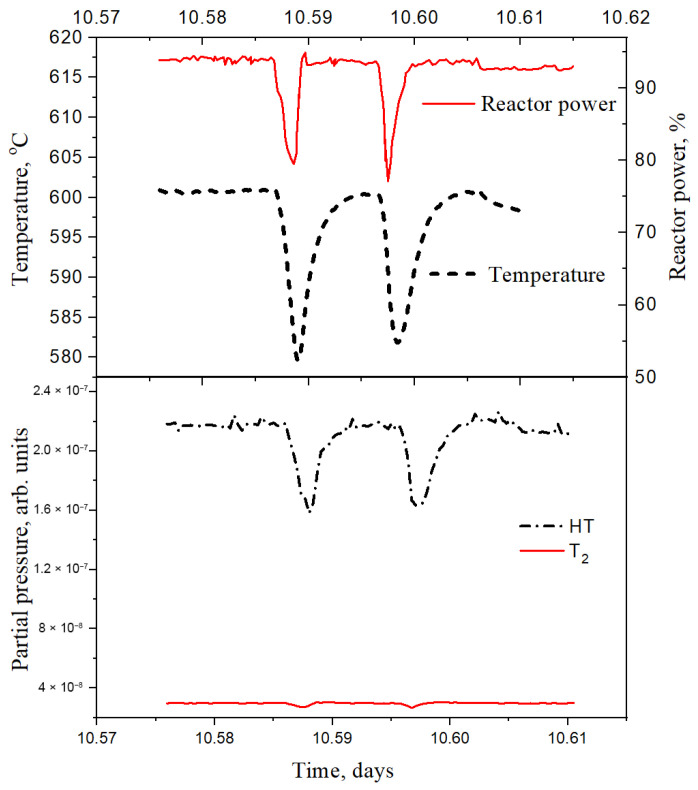
Experimental data on the release of tritium molecules during a short-term power decrease (section on 10.57 day of irradiation).

Figure 3 shows the input experimental data on the change in reactor power (section on 10.57 day of irradiation), sample temperature, and tritium molecule fluxes from ceramic samples. As can be seen from the figure, a short-term drop (by ~20%) and subsequent increase in reactor power lead to corresponding changes in temperature (by ~35 °C) and fluxes of released tritium molecules (they also fall and then grow). Such dynamics emphasize the high sensitivity of tritium release to temperature changes even under short-term impacts.

**Figure 4:**
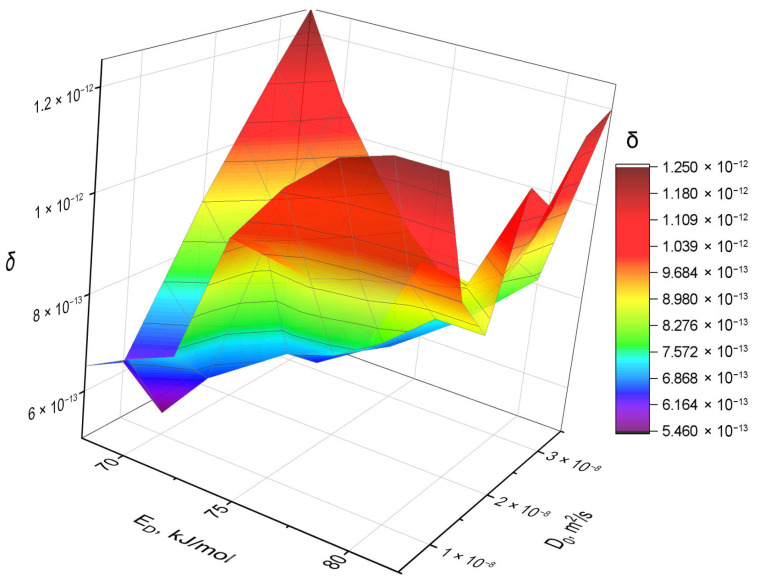
*δ*-function for various parameters *D*_0_ and *E_D_*, when modeling the experimental curve in the first section (10.57 days of irradiation).

**Figure 5:**
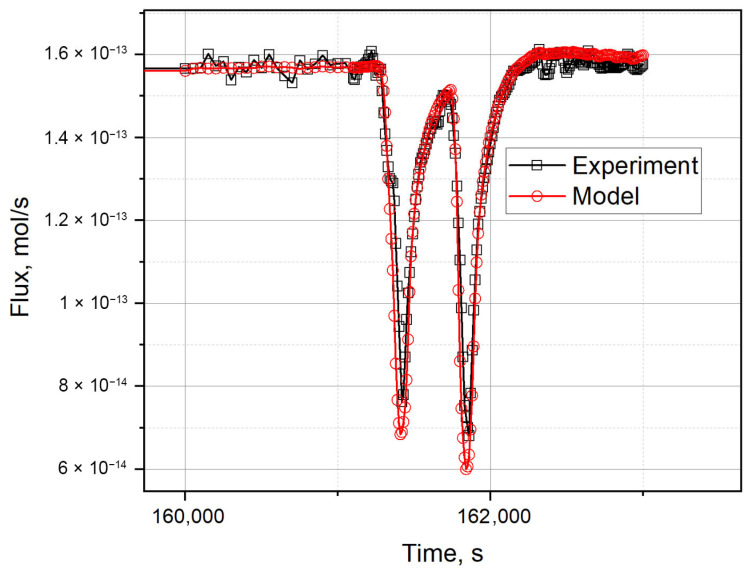
Results of tritium release simulation during a short-term power decrease (section on 10.57 day of irradiation) (*D*_0_~0.9 × 10^−8^ m^2^/s; *E_D_*~70.2 kJ/mol).

Figure 4 and Figure 5 show the simulation results for the first section of the reactor experiment. The values of the calculated *δ* function for different combinations of simulation parameters are shown in Figure 4, which shows the minimum corresponding to the variation parameters with the minimum value of the residual function. In Figure 5, the comparison of the model tritium release curve with the experimental data is presented. As can be seen, there is a good agreement between the simulated and experimental curves: the model perfectly reproduces both the absolute flow level and its dynamics with temperature changes. The coincidence in the shape of the curve and in the amplitude confirms the adequacy of the chosen approach and the applicability of the model to the analysis.

Based on the data obtained from the simulation, we can make reasonable assumptions about the sensitivity of the tritium output flux to variations in the main model parameters. The pre-exponential of the diffusion coefficient *D*_0_ affects the flux linearly: when *D*_0_ changes by ±10%, the flux changes in approximately the same way. The activation energy *E_a_*, which is part of the exponent of the Arrhenius expression, has a stronger effect. At a temperature of about 300 °C, a change of ±1 kJ/mol leads to a change in the flux by 7–10%, and at ±5 kJ/mol already by 30–40%. The flux is also extremely sensitive to temperature: with a temperature increase of 10 C, the diffusion coefficient can increase by 20–25%, which directly affects the tritium flux. This means that even small uncertainties in the temperature profile can significantly affect the simulation result. Geometrical parameters, such as the area of thermal contact between the pebble and the capsule base, also have an effect due to a change in the temperature gradient in the sample. Reducing the contact area increases the local temperature gradient, especially in the center of the pebble, and can reduce local diffusion coefficient D(T) and hence the flux. When the contact area is reduced by ~25%, the flux can decrease by 10–15%. Thus, the model is most sensitive to temperature, then to *E_a_*, and to a lesser extent to *D*_0_. Overall, this means that the model is robust to small deviations, but requires careful selection of the associated parameters.

## 4. Discussion

**Figure 6:**
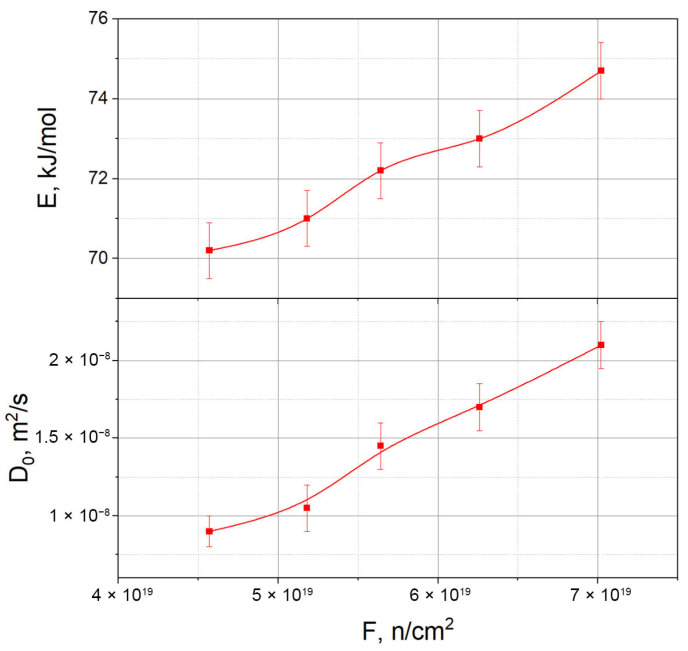
Calculated parameters of the Arrhenius dependence of tritium diffusion coefficients in lithium ceramics Li_2_TiO_3_ as a function of the thermal neutron fluence.

**Figure 7:**
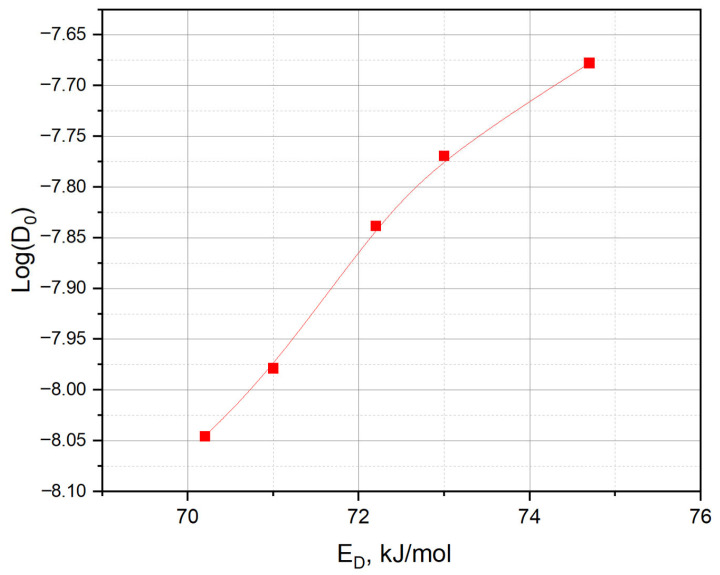
Dependence of the pre-exponential factor *Log*(*D*_0_) on the activation energy for tritium diffusion in lithium ceramics Li_2_TiO_3_.

The results of the simulation are shown in Figure 6 and Figure 7, and they indicate a slight increase in the activation energy depending on the thermal neutron fluence, as well as a noticeable rise in the pre-exponential factor *D*_0_.

It is worth noting that the dependence between *D*_0_ and *E_D_* (Figure 7) observed in the experiments can be represented by the Meyer–Neldel rule (MNR), also known as the compensation law, which means the linear relationship between the pre-exponential coefficient and the activation energy in thermally activated processes [27,28]. Such a dependence indicates the presence of multiple migration paths of tritium with different activation energies. In the case of tritium in irradiated lithium ceramics, this is due to the formation of radiation defects (vacancies, dislocations, and microcracks), which leads to a wide distribution of traps and trap types.

In [29], it was shown that the behavior of tritium in ceramics under the influence of irradiation changes significantly due to interactions with various defects, which vary slightly in parameters across different ceramics. In particular, specifically, in Li_2_TiO_3_, neutrons generate so-called F^+^ centers (oxygen vacancies with a captured electron) and O^−^ centers (electron-depleted oxygen atoms) [30]. At the same time, lithium burns out in tritium generation reactions, which leads to the formation of lithium vacancies in the lattice [24]. Together, these point defects (O^2−^ and Li^+^ vacancies, interstitial ions, etc.) act as capture centers for tritium, significantly hindering its migration.

Besides point defects, gaseous reaction products also play a significant role–mainly helium, which, like tritium, forms in the reaction ^6^Li(n,α)T. Helium remaining in the ceramics can create bubbles and pores, leading to swelling and cracking of the material. As shown by the gas release analysis in the current experiment [19], characteristic “peaks” of helium release were observed, corresponding to the opening of internal pores in the ceramics. These emissions primarily occurred within the first days of irradiation, when radiation heating and pressure buildup in large pores caused them to open. Pores of approximately 10 μm, 2 μm, and 1 μm in size opened successively (i.e., the larger one cracks earlier) over the first 7 days.

The cracks and channels formed during this process allowed for subsequent gas migration. Regarding the effect on tritium release, crack formation can reduce the diffusion path and enable faster release of some tritium through rapid diffusion pathways (via damage and pores). At the same time, microcracks create new surfaces and additional adsorption sites, which can hold tritium as compounds (for example, hydroxides) at higher temperatures.

Thus, the opposing effects of radiation damage—the formation of traps (vacancies, defects) and quick desorption pathways (cracks, pores)—together shape the overall kinetics of tritium release from irradiated ceramics.

In the calculation work presented, the sections of the experiment after the open porosity development of the samples were analyzed. So, generally, this effect was not incorporated into the modeling.

To compare the obtained results with the available literature data, we start by examining the calculated tritium diffusion parameters in ceramics, specifically for the initial stage of the current experiment, during the reactor’s gradual ramp-up phase [18] (Table 2).

The calculated diffusion coefficient values obtained in this study are generally similar to those reported earlier. However, the current work offers more precise estimates, as evidenced by lower uncertainties for both the activation energy and the pre-exponential factor of tritium diffusion. This improved accuracy mainly results from the modeling approach in [18], where both the diffusion coefficient and the desorption rate parameters were adjusted simultaneously, thereby reducing the uncertainty in determining each individual parameter.

A comparison with previously reported activation energies for tritium diffusion, obtained from experiment sections involving short-term power reductions [14], shows that these values were significantly overestimated compared to the current study’s results. This difference arises from the simplified phenomenological approach used earlier, which failed to consider critical factors such as the actual sample temperature, changes in tritium production rate during power reduction, and the assumption of a constant tritium concentration gradient near the sample surface.

As for the work [13], the diffusion parameters obtained in it cover a wide range of pre-exponential factors (10^−11^–10^−7^ m^2^/s) and activation energies (70–100 kJ/mol). Such a spread is due to the use of post-traditional TDS experimental techniques without a clear separation of diffusion and desorption processes. In addition, the reactor experiments were carried out at a significantly lower neutron fluence (<10^19^ n/cm^2^), where the effect of radiation-induced defects on diffusion is minimal. In the work [15], the values *D*_0_ = 5.8 × 10^−9^ m^2^/s and *E_a_* = 86 kJ/mol were also obtained for Li_4_SiO_4_ ceramics using the TDS method. The increased activation energy is probably due to the presence of surface and volume traps, as well as the high density and structural specificity of Li_4_SiO_4_, which differs from Li_2_TiO_3_. In addition, the data were obtained under conditions without reactor irradiation and reflect the combined contribution of diffusion and desorption, without taking into account radiation effects. Thus, the differences in the diffusion parameters given in Table 2 are associated with both the assessment methodology and the experimental conditions. The values obtained using simplified phenomenological models [3,13,15] do not take into account temperature gradients, tritium generation, and the effect of radiation defects, which leads to overestimated or scattered diffusion parameters. In contrast, this study uses a comprehensive model based on in situ experiments, which made it possible to obtain consistent and more reliable parameters reflecting the evolution of the material under irradiation.

Thus, the obtained values of the activation energy and the pre-exponential factor *D*_0_ are within the range of literature data, but show a clear increasing trend as the fluence accumulates, as recorded by systematic calculations

## 5. Conclusions

In this paper, we simulated tritium transfer processes in lithium ceramics Li_2_TiO_3_ under reactor irradiation using a previously developed comprehensive model that incorporates heat and mass transfer, tritium generation, as well as tritium diffusion and desorption. Based on modeling five sections of the experiment, corresponding to different thermal neutron fluences, we determined the parameters of the Arrhenius dependence of the tritium diffusion coefficient. The activation energy ranges from 70.2 to 74.7 kJ/mol, and the pre-exponential factor *D*_0_ varies from 9.0 × 10^−9^ to 2.1 × 10^−8^ m^2^/s. These values align with literature data obtained through both in-situ and offline experiments. However, unlike most studies, a stable trend showing the growth of activation energy and pre-exponential factor with increasing fluence is observed for the first time, indicating the influence of accumulated radiation damage on tritium diffusion kinetics.

The observed changes are explained by the evolution of the ceramic structure: accumulation of vacancies, pores, changes in microporosity, and tritium generation conditions. A linear dependence was established between *D*_0_ and *E_a_*, corresponding to the Mayer–Neldel rule and indicating the multiplicity of energy migration paths. The obtained results confirm the applicability of an integrated approach to the analysis of in situ experiments, the sensitivity of parameters to the accumulated dose, and the importance of taking into account radiation defects in modeling. They can be used to refine the parameters of calculation codes and plan future experiments.

## Figures and Tables

**Table 1:** Description of the simulation sections and their calculation results.

Simulation Area					
Time since the start of irradiation, days	10.57	12.12	13.05	14.51	16.25
Thermal neutron fluence since the start of irradiation, n/cm^2^	4.57 × 10^19^	5.18 × 10^19^	5.64 × 10^19^	6.26 × 10^19^	7.02 × 10^19^
Power change in the section, %	~20	~40	~20	~60	~50
Temperature change in the section, K	~35	~50	~35	~100	~80
Number of short-term power drops in the section	2	2	2	2	2
Calculated Parameters of the Diffusion Coefficient					
*D*_0_, m^2^/s	(9.0 ± 1.0) × 10^−9^	(1.1 ± 0.1) × 10^−8^	(1.5 ± 0.1) × 10^−8^	(1.7 ± 0.1) × 10^−8^	(2.1 ± 0.2) × 10^−8^
*E_D_*, kJ/mol	70.2 ± 0.7	71.0 ± 0.7	72.2 ± 0.7	73.2 ± 0.7	74.7 ± 0.7

**Table 2:** Comparison of diffusion parameters with literature data.

*D*_0_, m^2^/s	*E_D_*, kJ/mol	Comments	Reference
5.8 × 10^−9^	86	T in Li_4_SiO_4_, fluence less than 10^−19^ n/sm^2^	[31]
10^−11^–10^−7^	70–100	T in Li_2_TiO_3_, fluence less than 10^−19^ n/sm^2^	[30]
(3 ± 1) × 10^−8^	(72 ± 5)	T in Li_2_TiO_3_, fluence less than 10^−19^ n/sm^2^	[18]
-	82–87	T in Li_2_TiO_3_, fluence from 4 × 10^19^ n/sm^2^ to 8 × 10^19^ n/sm^2^ (simplified calculation method)	[14]
(0.5–2.1) × 10^−8^	70–75	T in Li_2_TiO_3_, fluence from 4 × 10^19^ n/sm^2^ to 8 × 10^19^ n/sm^2^ (complex simulation)	This study

## Data Availability

The original contributions presented in this study are included in the article. Further inquiries can be directed to the corresponding author.
